# Human Antibody Response to *Aedes albopictus* Salivary Proteins: A Potential Biomarker to Evaluate the Efficacy of Vector Control in an Area of Chikungunya and Dengue Virus Transmission

**DOI:** 10.1155/2014/746509

**Published:** 2014-04-13

**Authors:** Souleymane Doucoure, François Mouchet, Sylvie Cornelie, Papa Makhtar Drame, Eric D'Ortenzio, Jean Sébastien DeHecq, Franck Remoue

**Affiliations:** ^1^Institut de Recherche pour le Développement (IRD), Maladie Infectieuse et Vecteurs, Ecologie, Génétique, Evolution et Contrôle (MIVEGEC), UM1-CNRS 5290-IRD, Centre IRD de Montpellier, 34394 Montpellier, France; ^2^URMITE 198 Campus IRD-UCAD, Route des Pères Maristes, BP 1386, 18524 Dakar, Senegal; ^3^Agence Régionale de Santé, Océan Indien, CS 61002, 97743 Saint Denis Cedex 9, La Réunion, France; ^4^Agence Régionale de Santé, Océan Indien, CS 60050, 97408 Saint Denis Cedex 9, La Réunion, France

## Abstract

*Aedes* borne viruses represent public health problems in southern countries and threat to emerge in the developed world. Their control is currently based on vector population control. Much effort is being devoted to develop new tools to control such arbovirus. Recent findings suggest that the evaluation of human antibody (Ab) response to arthropod salivary proteins is relevant to measuring the level of human exposure to mosquito bites. Using an immunoepidemiological approach, the present study aimed to assess the usefulness of the salivary biomarker for measuring the efficacy of *Ae. albopictus* control strategies in La Reunion urban area. The antisaliva Ab response of adult humans exposed to *Ae. albopictus* was evaluatedbefore and after vector control measures. Our results showed a significant correlation between antisaliva Ab response and the level of exposure to vectors bites. The decrease of *Ae*. *albopictus* density has been detected by this biomarker two weeks after the implementation of control measures, suggesting its potential usefulness for evaluating control strategies in a short time period. The identification of species specific salivary proteins/peptides should improve the use of this biomarker.

## 1. Introduction

*Aedes albopictus* is one of the most invasive mosquito species in the world [1] and transmits a number of pathogens affecting humans particularly (re)emerging arboviruses, such as chikungunya and dengue virus [2]. In Asia, Africa, South America, and the Pacific, these arthropod-borne diseases are considered to be public health problems [3] while they threat to emerge in the developed world [4]. So far, no curative or prophylactic treatment is available to fight these emerging arboviruses. The only strategy for controlling their transmission is currently based on vector control, especially on interventions that aim to reduce the human-vector contact. Three types of interventions can be implemented: (i) to strengthen the population awareness about the risk of arbovirus transmission, (ii) to reduce the larval breeding sites by the elimination of water-holding containers and/or by using larvicides, and (iii) to control the local adult mosquitoes by insecticides spraying. This last strategy can prevent urban districts from outbreak [5]. The evaluation of the efficacy of these interventions is crucial for an optimal control of arboviruses transmission. This evaluation is currently based on the classical entomological methods, such as the identification of positive breeding sites, the capture of mosquitoes by traps, indoor spraying, and human landing catches techniques [6, 7]. The indices of Breteau, Adult Productivity, and House and Adult density are the most commonly used indicators for evaluating the abundance of* Aedes *population [8]. However, these entomological methods present considerable limitations when it comes to measuring the level of human exposure to anthropophilic* Aedes*. For example, the count of positive breeding sites is a very long and fastidious method to obtain solid results and the evaluation of adult* Aedes *density by usual entomological methods is not sensitive enough to estimate the low-level exposure to vector bites. These current methods are mainly applicable at the population level (e.g., at the household level) and are not able to evaluate the heterogeneity of individual exposure to* Aedes *bites. Altogether, these methods have substantial limitations in terms of large-scale field application and may raise ethical concerns, especially for human landing catches. In addition, due to a differential individual attractiveness to mosquitoes [9, 10] or other environmental and socioeconomic factors, these methods are not adapted to consider the heterogeneity of individual exposure to vector bites within the population. These limitations are even more considerable in the context of urban exposure. In order to improve the vector control and the survey of the risk of arbovirus transmission, many efforts are being devoted to develop new, simple, rapid, and highly sensitive complementary indicators to evaluate the level of human exposure to* Aedes *bites and the efficacy of control strategies. Recent findings suggested that the human antibody (Ab) response to vector salivary proteins, injected in human skin during the bite, can be a relevant immunoepidemiological marker to evaluate human exposure to vector bites. Indeed, individuals in contact with the vectors' bites could produce various levels (high to low) of antisaliva Ab response which depend on the real level of human exposure to vectors bites. This evidence has been demonstrated for a wide range of vectors, such as tick [11–14], mosquito [15–19], and sand flies [20–23]. Furthermore, this biomarker approach has been used to evaluate the efficacy of vector control strategy employed in the field by the quantitative evaluation of antisaliva immunoglobulin G (IgG). Indeed, the comparison between the initial level of antisaliva IgG (before the implementation of the vector control strategy) and the level of specific IgG, observed weeks or months after the intervention, has given substantial indications on the efficacy of the strategy used to control the population of vectors. For example, recent studies on malaria and chagas diseases have shown that the decrease of vector densities observed after the implementation of insecticide treated nets coincided with the decrease of the antivector saliva IgG response, both at individual and community level [24–26]. Concerning* Aedes*, the evaluation of antisaliva IgG response could give a measure of human exposure to biting vectors [15, 27]. In particular, we have shown that IgE and IgG4 isotype responses to* Ae. aegypti* saliva could be detected in young African children during the exposure season [28]. More recently, it has been shown that the IgG Ab level to whole saliva was associated with the intensity of exposure to* Aedes*, measured by the referent entomological indicators in urban areas in Bolivia [29]. In addition, it has been shown that adult populations in Reunion Island, where inhabitants are strongly exposed to* Ae. albopictus* bites, developed a high IgG response to the whole saliva of this vector, and very low cross-reactivity with* Ae. aegypti *salivawas observed in this context [30]. However, no previous study has been focused on the possibility of using the antisaliva IgG response for monitoring the effectiveness of control strategies targeting specifically* Aedes* vectors.

The present study addresses the potential application of such salivary biomarker as a complementary indicator to evaluate the efficacy of one* Ae. albopictus *vector control strategy based on insecticide space spraying and the elimination of all positive breeding sites. The main objective was to evaluate the IgG response to* Ae. albopictus* whole salivary gland extracts (SGE) in adult human populations before and after the initiation of the control measures in urban areas of Reunion Island. The vector control efficacy was especially evaluated during a short-time period, that is, two, four, and six weeks after interventions. The immunological results were compared with the entomological data, used as references, and the rainfall records during the studied period.

## 2. Materials and Methods

### 2.1. Ethics Statement

This study followed the ethical principles as stipulated in the Edinburgh revision of the Helsinki Declaration. The protocol was approved by a French Ethics Committee (the* Sud Ouest, Outre Mer* Ethics Committee, 25/02/2009) and authorized by French Drug Agency (AFFSAPS, Ministry of Health, 12/01/2009). Written informed consent was obtained from all subjects included in the study.

### 2.2. Studied Population

The study was carried out in “Le Chaudron (20°5′60′′N and 55°30′0′′E, 106 m asl) and “Les Camelias” (20°52′0′′N and 55°28′0′′E, 113 m asl), two urban districts of Saint Denis, the biggest city of Reunion Island. Chikungunya transmission was high during the 2006 epidemic. Seventy-five households were randomly selected and 101 individuals, from 18 to 65 years of age, were included for a longitudinal follow-up during the seasonal peak of Ae. albopictus abundance, from the 2nd of May to 9th of July 2010. The population was arbitrarily divided into three age groups: 18–35 years' group (33 individuals), 36–50 years' group (34 individuals), and the >50 years' group (34 individuals). The evaluation occurred before T0 at two (T2), four (T4), and six (T6) weeks after vector control intervention performed by ARS (Agence Régionale de Santé) technicians. At each visit, dried blood spots (on filter paper) were collected from each individual for immunological analysis. Just after the T0 visit, the vector control strategies were implemented and consisted of two combined strategies: (i) the physical elimination of all positive breeding sites (i.e., presence of* Ae. albopictus* larvae) by ARS entomological team and (ii) the treatment by two deltamethrin space sprays at 1 g/ha, at two days' interval, in the study area. At each following passage (T2, T4, and T6), all* Ae. albopictus* positive breeding sites were identified and also physically eliminated by the ARS team. Before their elimination at T0 and at T2, T4, and T6, the larval indices were calculated as follows: (i) the House index (HI): the percentage of houses infested with larvae and/or pupae and (ii) the Breteau index (BI): the number of positive containers per 100 houses. During the follow-up, the densities of adult* Ae. albopictus *were monitored every two days by using four Mosquito Magnet traps baited with CO_2_ and octenol.

Sera of unexposed individuals (*n* = 18) from a region free of* Ae. albopictus* and* Ae. aegypti* (north of France) were used as a negative control.

### 2.3. Collection of* Aedes* Salivary Gland Extracts (SGE)

SGE were obtained from 10-day-old uninfected females bred in insectary.* Ae. albopictus* strain was bred from larvae collected in the field in Reunion Island (Agence Régionale de Santé, Saint Denis, Reunion Island). Briefly, two days after a blood meal, the mosquitoes were sedated with CO_2_, and the salivary glands were dissected out and transferred into a tube containing 30 *μ*L of phosphate buffer saline (PBS). The dissected glands were then pooled in 30 or 60 pairs per batch and frozen at −80°C before extraction. A simple technique consisting of 3 successive freeze-thaw cycles in liquid nitrogen was used to disrupt the membranes. The soluble SGE fraction was then separated by centrifugation for 20 minutes at 30,000 g (at +4°C). The quantity of protein was evaluated by the Bradford method (OZ Biosciences) after pooling of the different batches to generate a homogenous SGE for immunological assessment. SGEs were then stored at −80°C before use.

### 2.4. Evaluation of Human IgG Ab Levels

An enzyme-linked immunosorbent assay (ELISA) was carried out using Maxisorp plates (Nunc, Roskilde, Denmark) coated with Ae. albopictus SGE (2 *μ*g/mL in phosphate buffer saline (PBS)) at 37°C for 150 min. Plates were blocked using 250 *μ*L of protein-free Blocking-Buffer (Pierce, Thermo Fisher, France) for 60 minutes at room temperature. Individual sera were incubated in duplicate at a 1/100 dilution in PBS-Tween 1%, 4°C overnight. Monoclonal mouse biotinylated Ab against human IgG (BD Pharmingen, San Diego, CA) was incubated at a 1/1000 dilution for 90 minutes at 37°C. Peroxidase-conjugated streptavidin (GE, Orsay, France) was added at 1/1000 for 60 minutes at 37°C. Colorimetric development was carried out using ABTS (2,2′-azino-bis (3-ethylbenzthiazoline 6-sulfonic acid) diammonium) in 50 mM citrate buffer (pH 4) containing 0.003% H_2_O_2_, and absorbance was measured after 120 min at 405 nm. Each test sample was assessed in duplicate wells and in a blank well without antigen (OD_*n*_) to measure nonspecific reactions. Individual results were expressed as the ΔOD value calculated using the equation ΔOD = OD_*x*_ − OD_*n*_, where OD_*x*_ represents the mean of the OD readings in the two antigen wells. An individual was considered as an “immune responder” if the ΔOD result was higher than the mean ΔOD+ (3 SD) for unexposed individuals (negative control). The ΔOD threshold of immune response was 0.5 for IgG Ab level to* Ae. albopictus*.

### 2.5. Statistical Analysis

GraphPad Prism Software (San Diego, CA, USA) was used to analyse the data. After confirmation of nonnormal distribution, a nonparametric Mann-Whitney test was used to compare Ab levels between two independent groups, and a nonparametric Kruskal-Wallis test was used for comparisons between more than two groups. All differences were considered significant at *P* < 0.05.

## 3. Results

### 3.1. Entomological Data and Rainfall

**Figure 1:**
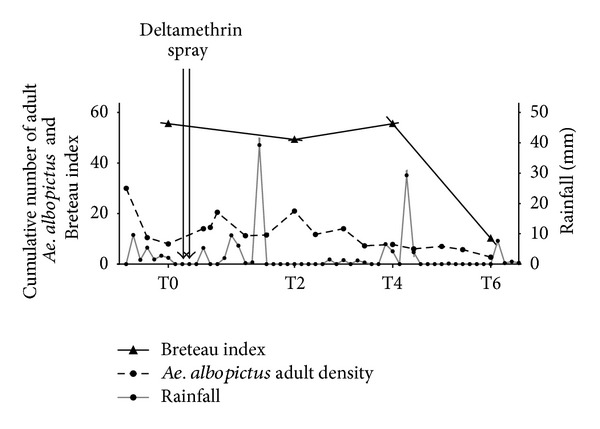
Evolution of entomological indices and rainfall for the studied period, Reunion Island, 2010. The entomological data of exposure to Ae. albopictus were presented as density of Ae. albopictus adult population (dotted line) and Breteau index (solid line, dark triangle) in the studied households. The daily records of rainfall intensity in the studied site (grey line, dark point) are presented according to the time-points. The timing of vector control intervention (deltamethrin spray) is indicated.

The presence of Aedes albopictus mosquitoes was observed through the Breteau index (BI) and House index (HI) during the follow-up period (Figure 1, Table 1). Most of the positive breeding sites were artificial and constituted by saucers (48%) and small containers (40%). It indicated that the most of the Ae. albopictus breeding sites appeared to be “human made.” The number of houses with positive breeding sites (HI) and the number of positive breeding sites decreased progressively from T0 (23.4% before vector control intervention) to T6 (10% six weeks after vector control intervention). The number of positive houses was 38, 20, and 12 at T0, T2 (two weeks after vector control intervention), and T4 (four weeks after vector control intervention), respectively, and the number of positive breeding sites was 90, 36, and 20 at T0, T2, and T4, respectively. A slight decrease of positive houses is noticed two weeks after mosquito control measures implementation, while at the same time, a considerable reduction of the number of positive breeding sites is observed. At T6, this decrease is much pronounced with only 5 positive breeding sites in 5 houses (Table 1). The evaluation of* Ae. albopictus* adult densities showed a peak at T2 (Figure 1). The amount of rainfall dropped from 39.25 mm, five days before T2, to 0 mm at T2. In contrast to T2, a peak of rainfall was observed at T4, while adult* Ae. albopictus* densities decreased and remained low. This decrease of* Ae. albopictus* adult densities appeared to be more marked at T6 which is associated with a weak rainfall intensity. The BI decreased only at T6, whereas no difference was observed for T2 and T4 compared to T0 (Figure 1, Table 1). These results suggest that it appeared to be difficult to observe a clear relationship between the rainfall intensity and the densities of adult Ae. Albopictus, whereby breeding sites in La Reunion urban area were essentially linked to the behaviour of the local population.

### 3.2. Individual IgG Response to* Ae. albopictus* Saliva before and after Mosquito Control Measures

**Figure 2:**
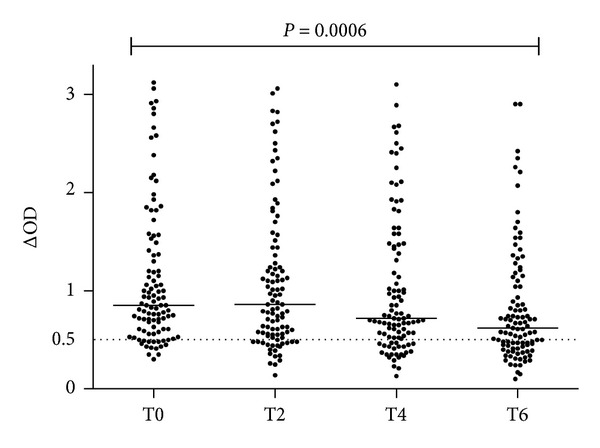
Evolution of IgG response to Ae. albopictus SGE before and after vector control intervention, Reunion Island, 2010. Inhabitants of two districts of Saint Denis city were visited just before T0 and then 2, 4, and 6 weeks after vector control. Individual IgG Ab responses are represented by ΔOD. Bars indicate median value and the dotted line represents the threshold of positivity of specific Ab response to Ae. albopictus SGE (cut-off ΔOD = 0.5).

The percentage (%) of immune responders (individuals with positive antisaliva IgG response) and the individual antisaliva IgG level (Figure 2) were compared before (T0) and after the vector control strategies at T2, T4, and T6 time-periods. For T0, T2, and T4 visits, the percentage of immune responders was not different with 86%, 80%, and 77%, respectively. No difference in specific IgG level was observed at T2 and T4 compared to T0. In contrast, a significant decrease of the percentage of immune responders was observed at T6 (65%) compared to T0 (P = 0.0006). This trend was also observed for the level of antisaliva IgG level. Indeed, despite the intraindividuals variation, no difference was observed between the 3 first visits with median values at 0.850, 0.860, and 0.720, respectively. A significant drop of specific IgG level was only observed at T6 (median value = 0.620) compared to T0 (*P* = 0.0006). This decrease of specific IgG at T6 was also significant when compared to T2 and T4 periods (*P* = 0.0018 and 0.0328, resp.). Therefore, at the whole population level, a significant decrease of the number of positive individuals (immune responder) and the level of the antisaliva IgG was only observed at T6 period.

**Figure 3:**
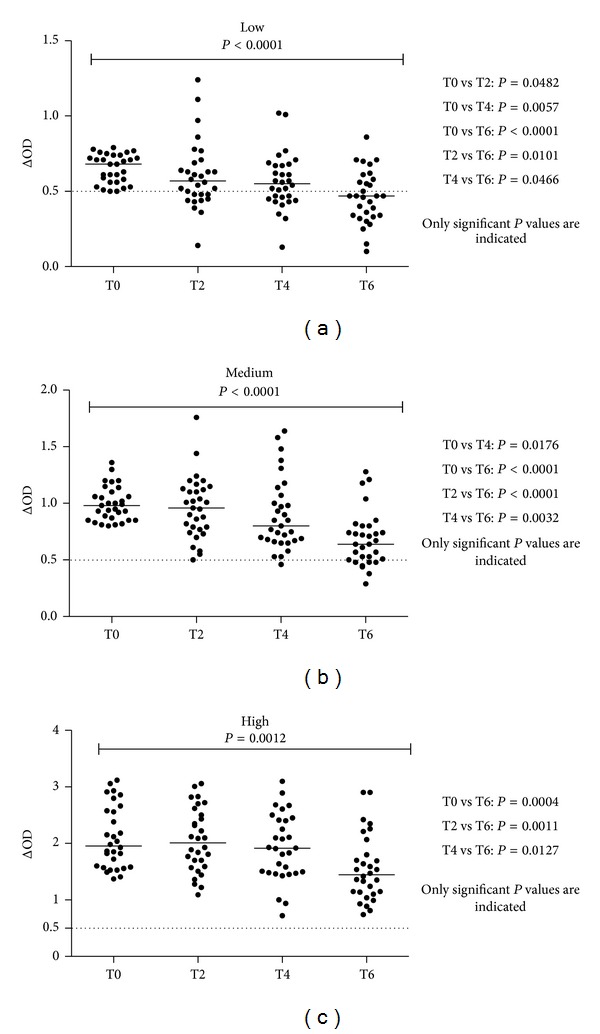
Evolution of individual IgG response to* Ae*.* albopictus* SGE according to initial Ab level, Reunion Island, 2010. The results of antisaliva IgG Ab level between T0 and T6 were presented for low (a), medium (b), and high (c) groups of “immune responders.” Bars indicate median value in each group and the dotted line represents the threshold of specific Ab response to* Ae*.* albopictus* SGE (ΔOD = 0.5). For all groups, the Kruskal-Wallis indicated significant difference between the different time periods: *P* < 0.0001, *P* < 0.0001, and *P* = 0.0012 for low, medium, and high groups, respectively. In the “low” group (a), test showed significant difference in T0 versus T2 (*P* = 0.0482), T0 versus T4 (*P* = 0.057), T0 versus T6 (*P* < 0.0001), T2 versus T6 (*P* = 0.0101), and T4 versus T6 (*P* = 0.0466); in “medium” group (b): T0 versus T4 (*P* = 0.0004), T0 versus T6 (*P* < 0.0001), T2 versus T6 (*P* < 0.0001), and T4 versus T6 (*P* = 0.0032); in “high” group (c): T0 versus T6 (*P* = 0.0004), T2 versus T6 (*P* = 0.0011), and T4 versus T6 (*P* = 0.0127).

The potential impact of this vector control strategy on antisaliva IgG level was assessed according to the initial level of specific IgG at T0 (before interventions). For these reasons, the population of study was divided into groups of immune responders according to their level of specific IgG response, defined by the cut-off value (ΔOD = 0.5) and the values of the tertiles initially observed at T0 (tertile 1: ΔOD = 0.796 and tertile 2: ΔOD = 1.363). Therefore, three groups of immune responders were defined as follows: low (0.50 ≤ ΔOD ≤ 0.796; *n* = 30), medium (0.796 < ΔOD ≥ 1.363; *n* = 31), and high (1.363 < ΔOD ≤ 3.12; *n* = 30). The evolution of the antisaliva IgG level in each group was checked during all the follow-up (T0 to T6, Figure 3). For the “low” group (Figure 3(a)), a significant decrease of antisaliva IgG level was observed from T2 (*P* = 0.0482 compared to T0). The antisaliva IgG level decreases then progressively from T2 to T6. This decrease was significant when comparing the T0 period to T4 (*P* = 0.0057) and to T6 (*P* < 0.0001). The percentage of immune responders also decreased progressively from T0 to T6. The percentage of immune responders was 100%, 67%, 63%, and 40% at T0, T2, T4, and T6, respectively.

For medium (Figure 3(b)) and high (Figure 3(c)) immunological groups, a significant decrease of the level of specific Ab response was observed at T4 and T6 (*P* = 0.0176 and 0.0004, resp.) compared to T0. In the medium group, the percentage of immune responders was 100% and 98% at T2 and T4, respectively. A marked decrease of the percentage of immune responders (77%) was observed at T6. For the high immunological group, no decrease of the percentage of immune responders is noticed, whereas significant decrease of the specific IgG level was observed at T6 compared to T0 (*P* = 0.0004). These trends indicated that the decrease of the level of antisaliva IgG was dependent on the initial level of Ab. The decrease was therefore faster (2 weeks) when the initial level of IgG was low, whereas it was longer in time (4 to 6 weeks) if the level of antisaliva IgG was high at T0.

The random effect of age and sex on the antisaliva IgG response was analysed. The population was divided based on three classes of age: 18–35, 36–50, and >50 years old (data not shown). At T0 period, the percentage of immune responders was very high and similar between each group: 88%, 89%, and 83%, respectively. The level of specific IgG was also similar among the three groups of age. For each group of age, the evolution of the antisaliva IgG level was evaluated from T0 to T6. For 18–35 age group, no significant decrease was observed during the follow-up period with values of the medians at 0.850, 0.810, 0.740, and 0.640 for T0, T2, T4, and T6, respectively. For the 36–50 age group, the T6 period was marked by a significant decrease of IgG level compared to T0 (*P* = 0.0115) and the value of the median value was 1.025 at T0 and 0.695 at T6. The same trend is observed with the >50 age group (*P* = 0.0107) with median value at 0.800 and 0.520 at T0 and T6 periods, respectively. According to the gender of studied individuals, the percentage of males and females in the studied population was 41% and 59%, respectively. A similar decrease of the median of specific IgG level from T0 to T6 was observed between males and females (Table 1) suggesting that this Ab decrease was not sex dependent. In addition, statistical analysis indicated that no random effect between both studied districts was observed in the IgG response against* Ae. albopictus* SGE (data not shown).

## 4. Discussion

In the present study, the human Ab response to* Ae. albopictus* SGE was investigated in adult individuals before and after* Ae. albopictus *control operation in Reunion Island.

In the whole population, the percentage of immune responders was high before (T0) and two (T2) and four (T4) weeks after vector control. A significant decrease of the percentage of immune responders (positive individuals for antisaliva IgG response) was only observed at six weeks (T6) after the intervention. The same pattern of postintervention evolution was also observed for entomological data. The evaluation of immature and adults* Ae. albopictus* stages indicated high density of vector populations at T0 and T2, followed by a decrease at T4 and T6 periods. These results pointed out a possible association between the entomological data and antisaliva IgG Ab level. The percentage of immune responders and the level of specific IgG at each visit were associated with the intensity of exposure to* Ae. albopictus* bites, evaluated by entomological methods. These results emphasized the relevance of the evaluation of antisaliva IgG response as an immunoepidemiological marker for* Ae. albopictus* exposure. They are consistent with previous data indicating the potential to use human Ab response for evaluating the exposure to vectors bites [17, 20].

We addressed the potentiality to use the Ab response against* Ae. albopictus* salivary proteins for monitoring vector control strategies against* Ae. albopictus*. In this study, a significant decrease of* Ae. albopictus* population (immature and adults) was observed at T6 period which seemed to be associated with the drop of the antisaliva IgG response at the whole population level. Similar results have been reported according to the use of protection tools against* Leishmania* and malaria vectors [20, 24, 25]. It suggests that the application of deltamethrin space spraying combined with the physical elimination of positive breeding sites (i.e., containing* Ae. albopictus* larvae) has an impact on* Ae. albopictus* population densities six weeks after the implementation of vector control operation. This is all the more surprising when considering that deltamethrin is a powerful adulticide, and until now, no* Ae. albopictus* resistance to this insecticide was recorded in La Reunion. This could suggest the interference of extrinsic factors on the decrease of* Ae. albopictus* population. Indeed, days before the T6 period, a considerable drop of rainfall was observed which would imply a potential climatic effect on the collapse of* Ae. albopictus* adult population. However, whatever the cause involving vector control or vectors seasonality, the period of decline of* Ae. albopictus* adult and larvae population coincided with a significant drop of the level of IgG directed to* Ae. albopictus* saliva. It indicates the possibility to use the antisaliva IgG response for monitoring* Ae. albopictus* population. These results are consistent with previous studies which have shown that the human-specific IgG response can represent a genuine biomarker for monitoring vector control program.

In the objective to better understand the evolution of specific IgG response after vector control, the studied population was divided into three groups of immunological responders according to the specific antisaliva IgG level: low, medium, and high. Indeed, a recent study indicated that the level of antisaliva IgG is reflected by the intensity of human exposure to* Ae. aegypti* bites. Weak specific IgG response was associated with low level of exposure to vector bites and, inversely, with high IgG response [29]. It suggests the potential to define different groups of immune responders which may correspond to increasing level of human exposure to* Ae. albopictus* bites. For all groups of immunological responders defined in this study, the same trend of high Ab decrease was observed at T6 period compared to T0. Interestingly, for the group of low immune responders, a significant decrease of the specific IgG level was observed at T2 period, which is only two weeks after vector control intervention. Such decrease was observed at T4 for medium group and only at T6 for group of high immune responders. These results suggest that the time-dependent decrease of antisaliva IgG levels after intervention was clearly linked to the initial level of specific Ab levels (T0). Altogether, these results indicated that antisaliva IgG response is characterized by a very short half-life, that is, only two weeks, and therefore the specific level of IgG could rapidly decrease after the interruption of the exposure to mosquito bites. This particular property of antisaliva immune response is clearly in favour of the development of pertinent biomarker measuring the efficacy of vector control strategy in a short-time period after the intervention. Previous studies have shown the possibility to use the salivary biomarker for evaluating the efficacy of insecticide treated nets six weeks after their implementation. However, in this study, it is difficult to establish a direct link between the early decrease of the level of antisaliva Ab and the potential efficacy of vector control intervention as neither the BI nor the adult density did not decrease significantly at T2.

The present study indicated, for the first time, the possibility to use such biomarker for monitoring vector control program in the case of human exposure to* Ae. albopictus*. It is consistent with previous investigations that highlighted the usefulness of human or animal Ab response against salivary proteins in monitoring the efficacy of vector control strategies based on insecticide treatment or insecticide treated nets [20, 24–26]. In addition, it could provide a potential tool for evaluating the immediate impact of vector control program, whereas the current entomological methods present considerable limitations to indicate a rapid decrease in* Ae. albopictus* population densities. In many endemic areas,* Ae. albopictus* is found in high density and some improvement of such tools may be needed to detect the decrease of vector density earlier after the implementation of vector control. One hypothesis is that the evaluation could be improved by using appropriate Ab isotypes like IgE and IgG4. For example, the measure of IgG4 has been used to detect an intense exposure to Aedes bites [16]. In the present study, no difference was observed in the antisaliva IgG response according to different age groups at T0. After the deltamethrin application and elimination of positive breeding sites, a decrease of specific IgG response was observed for the age groups of 36–50 and >50 years of age at T6, as compared to T0, but not for 18–35 years group. It suggests that this biomarker could not be pertinent whatever the age of individuals. However, previous study showed that* Anopheles* salivary biomarker was pertinent for evaluating malaria vector control whatever the age of individuals, including children [24]. This demonstrates the necessity to consider a wider age range than the population selected in this study and particularly the youngest population, like children, who are generally more sensitive to arthropod-borne diseases. Other factors, like human behaviour, may also be investigated to elucidate this difference of response according to age groups. For instance, the potentiality that the individuals in this study could be exposed to* Ae. albopictus* bites in places other than households cannot be excluded. Indeed, there are frequent movements of the studied population through the districts of Saint Denis which are characterised by numerous* Ae. albopictus* breeding sites. In addition, it cannot be excluded that some individuals used personal protective measures against* Aedes* bites (coil, clothing, etc.) which could interfere with the observed immunological results.

Although* Ae. albopictus* represents the only* Aedes* species which is known to bite human individuals, we cannot dismiss, in the present study, the hypothesis of potential cross-reactivity of IgG response to other mosquitoes salivary proteins as long as some salivary proteins are genus shared. Salivary protein or peptide specific to* Ae. albopictus* should be thus identified to improve the usefulness of this potential tool. This perspective is currently under investigation as previously developed for malaria vectors [19]. This salivary peptide has shown high sensitivity to detect human-vector contact with* Anopheles*, and this peptide approach can be beneficial for the detection of IgG level in individuals highly exposed to* Ae. albopictus* bites.

## 5. Conclusion

These results represent a step for showing that the measurement of human Ab response to* Ae. albopictus* salivary proteins could provide a reliable biomarker for evaluating the efficacy of vector control. This study strengthens the general approach for using Ab response to salivary antigens as a biomarker of human exposure to mosquito bites. Nevertheless, to fully ascertain the capacity of this biomarker to evaluate control strategies, further studies should be conducted to clearly link the decrease of vector densities to vector control measures.

## Figures and Tables

**Table 1:** Entomological parameters and specific IgG Ab median level, Reunion Island, 2010.

	T0	T2	T4	T6
Number of visited houses	162	55	65	49
Number of positive breeding sites	90	36	20	5
House index*	23.4%	6.8%	18.5%	10.2%
Breteau index*	55.5	49.3	55.5	10.2
Total population IgG median	0.850 [0.300–3.120]	0.860 [0.140–3.060]	0.720 [0.130–3.100]	0.620 [0.100–2.900]
Males IgG median	0.740 [0.350–2.930]	0.770 [0.260–3.010]	0.650 [0.290–2.680]	0.550 [0.150–2.350]
Females IgG median	0.860 [0.300–3.120]	0.860 [0.140–3.060]	0.720 [0.130–3.100]	0.630 [0.100–2.900]

*House index (HI): the percentage of houses infested with larvae and/or pupae.

*Breteau index (BI): the number of positive containers per 100 houses.
